# Sn/Be Sequentially co-doped Hematite Photoanodes for Enhanced Photoelectrochemical Water Oxidation: Effect of Be^2+^ as co-dopant

**DOI:** 10.1038/srep23183

**Published:** 2016-03-23

**Authors:** Alagappan Annamalai, Hyun Hwi Lee, Sun Hee Choi, Su Yong Lee, Eduardo Gracia-Espino, Arunprabaharan Subramanian, Jaedeuk Park, Ki-jeong Kong, Jum Suk Jang

**Affiliations:** 1 Division of Biotechnology, Advanced Institute of Environmental and Bioscience, College of Environmental and Bioresource Sciences, Chonbuk National University, Iksan 570-752, Republic of Korea; 2 Pohang Accelerator Laboratory, POSTECH, Pohang 790-784, Republic of Korea; 3 Department of Physics, Umeå University, Umeå, SE-901 87, Sweden; 4 Center for Chemical Safety and Security, Korea Research Institute of Chemical Technology (KRICT) Daejeon, 305-343, Republic of Korea

## Abstract

For *ex-situ* co-doping methods, sintering at high temperatures enables rapid diffusion of Sn^4+^ and Be^2+^ dopants into hematite (α–Fe_2_O_3_) lattices, without altering the nanorod morphology or damaging their crystallinity. Sn/Be co-doping results in a remarkable enhancement in photocurrent (1.7 mA/cm^2^) compared to pristine α–Fe_2_O_3_ (0.7 mA/cm^2^), and Sn^4+^ mono-doped α-Fe_2_O_3_ photoanodes (1.0 mA/cm^2^). From first-principles calculations, we found that Sn^4+^ doping induced a shallow donor level below the conduction band minimum, which does not contribute to increase electrical conductivity and photocurrent because of its localized nature. Additionally, Sn^4+^-doping induce local micro-strain and a decreased Fe-O bond ordering. When Be^2+^ was co-doped with Sn^4+^-doped α–Fe_2_O_3_ photoanodes, the conduction band recovered its original state, without localized impurities peaks, also a reduction in micro-strain and increased Fe-O bond ordering is observed. Also the sequence in which the *ex-situ* co-doping is carried out is very crucial, as Be/Sn co-doping sequence induces many under-coordinated O atoms resulting in a higher micro-strain and lower charge separation efficiency resulting undesired electron recombination. Here, we perform a detailed systematic characterization using XRD, FESEM, XPS and comprehensive electrochemical and photoelectrochemical studies, along with sophisticated synchrotron diffraction studies and extended X-ray absorption fine structure.

Improvement of the electrical conductivity of semiconductor metal oxides is one of the most profound challenges in the development of high performance photoanodes for photoelectrochemical (PEC) water splitting^1^,^2^. Various semiconducting metal oxides like TiO_2_ 3, WO_3_ 4, CdWO_4_ 5 and α-Fe_2_O_3_ 6 have been studied for PEC water splitting. Hematite (α–Fe_2_O_3_) is considered an ideal metal-oxide semiconductor photoanode for PEC applications, owing to its stability, suitable band gap (2.2 eV), low cost and non-toxic nature^7^. However, pristine α–Fe_2_O_3_ exhibits poor performance due to short hole diffusion lengths (2–4 nm) and low electron mobility^8^. Doping of α–Fe_2_O_3_ photoanodes has been extensively investigated to improve its photoelectrochemical properties^9^. The introduction of mono-dopants (such as Sn^10^, Ti^11^, Si^12^, Pt^13^, Zr^14^, Ge^15^,^16^, Cr^17^ and Zn^18^) has been used to enhance the PEC performance of α–Fe_2_O_3_ photoanodes. Electron-donor dopants introduce electrons into neighboring Fe^3+^ sites and reduce Fe^3+^ to Fe^2+^ 19,20. Since the electrical conductivity of α-Fe_2_O_3_ follows the polaron hopping mechanism^7^, the newly formed Fe^2+^ sites can improve electrical conductivity with electron donor dopants such as Si^21^ and Sn^10^. The cationic elemental doping enhances conductivity as a result of increased donor concentration and improved charge transfer^8^. Sn is an effective dopant for α–Fe_2_O_3_ photoanodes, with an ionic radius and Pauling electronegativity similar to those of Fe ions^22^. Introduction of co-dopants generally improves the photoelectrochemical properties of α–Fe_2_O_3_ photoanodes. Recently, many research groups have reported that co-doping may significantly improve the PEC performance of α–Fe_2_O_3_ photoanodes through various mechanisms^23^,^24^,^25^,^26^,^27^,^28^. For example, the ionic radius difference between Fe^3+^, Si^4+^ and Ti^4+^ increases the donor concentration for Si and Ti co-doped α–Fe_2_O_3_ 24. N and Zn co-doped α–Fe_2_O_3_ possesses a higher concentration of acceptors, and exhibits improved photo-response with Zn doping and lower dark current with N doping^26^. Zn and Ti co-doped α–Fe_2_O_3_ enhances PEC device performance through increased electrical conductivity and improved charge transport properties^28^. When electron donor dopants such as Si^4+^, Ti^4+^ and Sn^4+^ are introduced into the α-Fe_2_O_3_ lattice, Fe^3+^ in the α-Fe_2_O_3_ lattice is replaced by the respective 4+ dopant cations^19^,^29^. Generally, *in-situ* doping methods alter the crystallinity and morphology of α–Fe_2_O_3_, which may have a substantial impact on PEC device performance^24^,^26^,^27^,^28^,^30^.

Here we discuss a simple *ex-situ* co-doping method, employing Sn and Be as dopants in a lattice of α–Fe_2_O_3_ nanorods. For the *ex-situ* co-doping method, Sn and Be were deposited onto α–Fe_2_O_3_ nanorods by dip-coating, followed by high temperature sintering (800 °C for 10 min). High temperature sintering and an ultrafast heating rate enable rapid dopant diffusion into the α–Fe_2_O_3_ lattice, and such brief 800 °C sintering minimizes morphology and crystallinity changes in the α–Fe_2_O_3_ nanorods. Introduction of Sn^4+^ mono-dopants into α-Fe_2_O_3_ photoanodes enhances their PEC properties by improving electrical conductivity and reducing transport resistance (by improving the photoanode/electrolyte interface)^30^. However, the Sn^4+^ mono-doping induces various undesirable changes in the α-Fe_2_O_3_ photoanodes, including a change in conduction band position and an increase in micro-strain compared to pristine photoanodes. However, the introduction of a secondary dopant, such as Be^2+^, along with Sn^4+^ dramatically enhances the PEC properties of α-Fe_2_O_3_ photoanodes from 0.7 mA/cm^2^–1.7 mA/cm^2^ at 1.23 V_RHE_. Here we present a detailed, systematic characterization of the role of Be^2+^ as an effective co-dopant using XRD, FESEM, XPS and detailed electrochemical and photoelectrochemical studies. Furthermore, we performed density functional theory (DFT) calculations to understand the doping effects induced on the structural and electronic properties. Structural information obtained by EXAFS and micro-strain analysis from the synchrotron XRD studies give a clearer picture of the micro- and macro- changes in the doped photoanodes. To the best of our knowledge, this is the first experimental demonstration of Sn^4+^ and Be^2+^ co-doping of α–Fe_2_O_3_ photoanodes employing an *ex-situ* co-doping method.

## Results & Discussion

**Figure 1:**
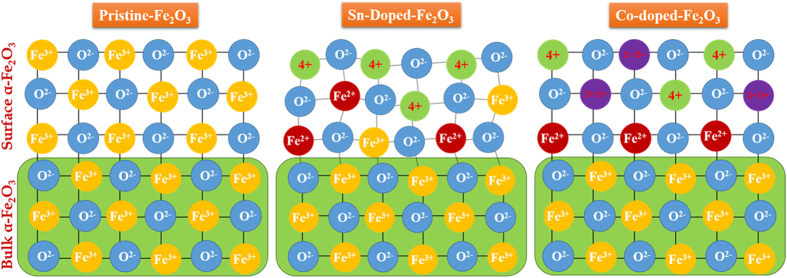
Schematic representation of Sn^4+^ dopants introducing the Fe^2+^ sites in α-Fe_2_O_3_ lattice while Be^2+^ dopants stabilizing the newly formed Fe^2+^ sites in α-Fe_2_O_3_.

The Sn^4+^ and Be^2+^ *ex-situ* co-doping method for α–Fe_2_O_3_ photoanodes is illustrated in the Fig. 1. First, β-FeOOH nanorods, average length of 400 nm, was grown on FTO substrates by a hydrothermal method followed by 550 °C sintering in order to promote a phase transition from β–FeOOH to pure α–Fe_2_O_3_. Afterwards, Sn^4+^ and Be^2+^ co-doping on α–Fe_2_O_3_ nanorods were carried out using a simple *ex-situ* doping method. The α–Fe_2_O_3_ nanorods were dip-coated with the Sn-precursor, dried by air blowing (leaving a thin layer of Sn precursor), then dipped into Be-precursor and dried once again. The Sn/Be-coated α–Fe_2_O_3_ nanorods were finally sintered at 800 °C for 10 min, promoting the effective diffusion of Sn^4+^ and Be^2+^ dopants into the α–Fe_2_O_3_ lattice.

**Figure 2:**
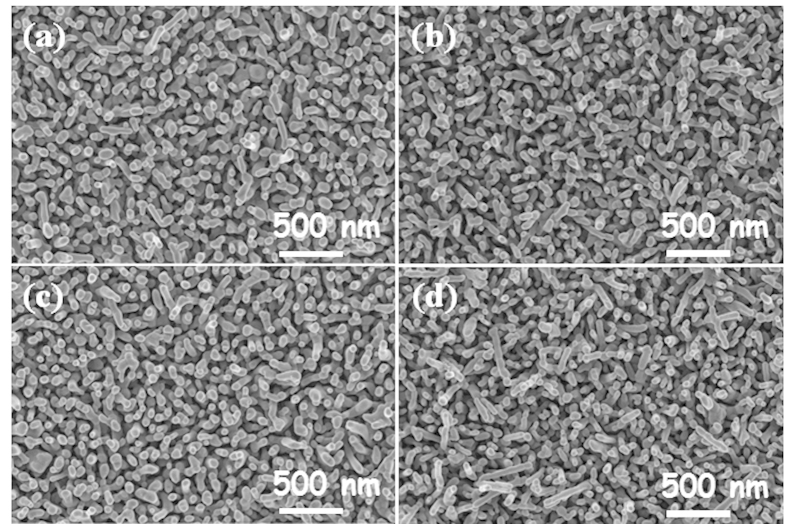
FESEM images of (**a**) pristine, (**b**) Sn-doped, (**c**) Be-doped and (**d**) co-doped α–Fe_2_O_3_ photoanodes sintered at 800 °C.

Figure S1 illustrates the XRD patterns of pristine, Sn-doped, Be-doped, and Sn/Be-doped α–Fe_2_O_3_ photoanodes. With the exception of FTO substrate peaks, all other peaks can be indexed to α–Fe_2_O_3_ (JCPDS card #33–0664). Aside from those of α–Fe_2_O_3_, no diffraction peaks of Sn, Be or other impurity phases were observed. All pristine and doped α–Fe_2_O_3_ photoanodes displayed similar diffractograms with a predominant (110) diffraction peak^10^,^31^. Figure 2 illustrates the FESEM images comparing pristine, Sn-doped, Be-doped, and co-doped α–Fe_2_O_3_ photoanodes. Both pristine and doped α–Fe_2_O_3_ photoanodes were sintered at 800 °C and show very similar nanorod morphology, with diameters of 30–50 nm and lengths of approximately 400 nm, roughly vertical to FTO substrates^13^. From the UV-vis spectroscopy measurements (Fig. S2), we determined that doping did not produce intermediate band levels within the bandgap of α–Fe_2_O_3_, since doping did not alter the bandgap of α–Fe_2_O_3_ photoanodes. Our *ex-situ*, sequential co-doping method has several advantages over *in-situ* or other physical vapor deposition methods that have been reported previously. Our *ex-situ* doping method involves rapid dopant diffusion at high temperature, which minimizes crystallinity and morphology changes in α–Fe_2_O_3_ photoanodes^32^,^33^,^34^. In order to confirm the incorporation of Sn^4+^ and Be^2+^ dopants into the α–Fe_2_O_3_ nanorods, X-ray photoelectron spectroscopy (XPS) analyses were performed on co-doped α–Fe_2_O_3_ photoanodes. Figure S3 displays the XPS spectra of the full survey scan, Fe *2p*, Sn *3d* and Be *1s* regions obtained from co-doped α–Fe_2_O_3_ nanorods. Fe *2p* spectra revealed that iron existed predominately as Fe^3+^, with binding energies for Fe2p_3/2_ and Fe2p_1/2_ levels located at 710.9 and 723.9 eV, respectively. These peaks are consistent with Fe^3+^ ions in α-Fe_2_O_3_ 35. We also observed two XPS peaks for Sn *3d* at 486.3 and 494.1 eV, corresponding to binding energies of Sn3d_5/2_ and Sn3d_3/2_ respectively^36^. Similarly, the presence of Be^2+^ dopants was supported by peaks around 113.5 eV, evidence of Be incorporation into the α–Fe_2_O_3_ photoanodes^37^. XPS (Table S1) and ICP analysis (Table S2) confirmed that Sn^4+^ and Be^2+^ were successfully co-doped into the α-Fe_2_O_3_ lattice with our *ex-situ* co-doping method.

**Figure 3:**
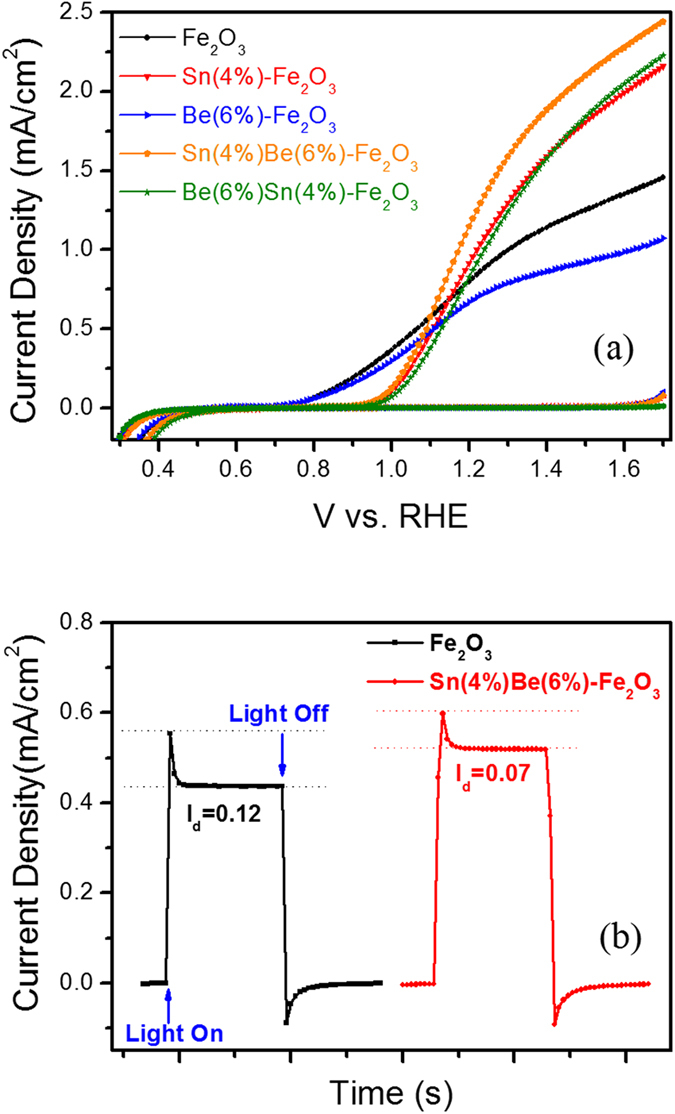
(**a**) Photocurrent-potential (*J–V*) curves and (**b**) Transient photocurrent measurement for PEC water oxidation reaction with pristine and doped α–Fe_2_O_3_ photoanodes under standard illumination conditions.

Figure 3(a) illustrates the PEC performance of pristine, Sn-, Be-doped and Sn/Be co-doped α–Fe_2_O_3_ photoanodes. The photocurrent density of Sn-doped and Sn/Be co-doped α–Fe_2_O_3_ photoanodes was dramatically improved compared to pristine α–Fe_2_O_3_ photoanodes. A photocurrent density of 0.75 mA/cm^2^ was observed at 1.23 V_RHE_ for pristine α–Fe_2_O_3_ photoanodes, and increased dramatically to 1 mA/cm^2^ for Sn-doped α–Fe_2_O_3_ photoanodes. The maximum photocurrent density, 1.7 mA/cm^2^, was exhibited by a Sn/Be co-doped photoanode with a dopant concentration of 4% and 6%, respectively, this photocurrent density is two times larger than that of pristine α–Fe_2_O_3_ photoanodes (see Fig. 3a and S4), proving that the incorporation of Sn^4+^ dopants into the α-Fe_2_O_3_ lattices enhances PEC device performance. We observed similar results for Sn^4+^ mono-doped α-Fe_2_O_3_ photoanodes with improved performance compared to pristine α-Fe_2_O_3_ 30. Surprisingly, incorporation of Be^2+^ as a mono-dopant does not enhance the photocurrent of pristine α-Fe_2_O_3_ photoanodes. However, when Be^2+^ is co-doped with Sn^4+^, there is a synergetic increase in photocurrent when compared with pristine and Sn^4+^ mono-doped α-Fe_2_O_3_ photoanodes. With Sn as a mono-dopant, Fe^3+^ ions will be replaced by the dopant Sn^4+^ ions, thereby increasing the electron carrier concentration and enhancing electron transport properties, in turn increasing PEC device performance.

From transient photocurrent measurements (Fig. 3b) at a constant potential (1.1 V_RHE_), the current decay (*I*_*d*_) (difference between initial current (*I*_*i*_) and final current (*I*_*f*_); *I*_*d*_ *= I*_*i*_ *− I*_*f*_) decreased from 0.12–0.07 mA/cm^2^ for co-doped photoanodes compared to pristine α–Fe_2_O_3_ photoanodes. IPCE analyses of doped and pristine α–Fe_2_O_3_ photoanodes were performed at various wavelengths, as shown in Fig. S5. The Sn-Be co-doped α–Fe_2_O_3_ photoanodes had the highest IPCE values (29% at 340 nm) at 1.4 V_RHE_. Both Sn-doped α–Fe_2_O_3_ photoanodes (25% at 340 nm) and pristine α–Fe_2_O_3_ photoanodes (17% at 340 nm) exhibited reduced IPCE values. In comparison to pristine α–Fe_2_O_3_ photoanodes, the co-doped α–Fe_2_O_3_ photoanodes exhibited excellent photochemical stability and photochemical response (Fig. S6). These IPCE results were consistent with the difference in photocurrent densities observed in pristine and doped α–Fe_2_O_3_ photoanodes. Sn^4+^ donor dopants in α-Fe_2_O_3_ lattices introduce electrons to Fe^3+^ sites, reducing Fe^3+^ to Fe^2+^ 19,20. These Fe^2+^ sites can thus improve the electrical conductivity of α-Fe_2_O_3_ photoanodes via the polaron hopping mechanism^10^. The substitutional Sn^4+^ dopant ions induce an extra positive charge on the α-Fe_2_O_3_ lattice which can be compensated by reducing Fe^3+^ to Fe^2+^ (preserving charge neutrality)^38^. In order to attain charge neutrality at the doping interface, it is necessary to incorporate another stable 2+ cation, such as Be^2+^ dopants^23^.

**Figure 4:**
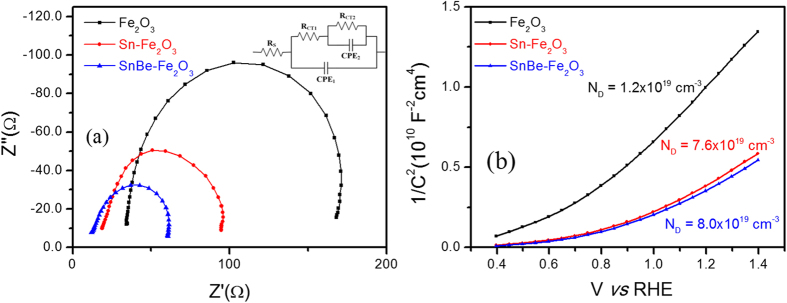
(**a**) Nyquist plots and (**b**) Mott-Schottky plots of pristine, Sn-doped and co-doped α–Fe_2_O_3_ photoanodes at 1.23 V_RHE_, under illumination conditions. The inset of Nyquist plot represents the equivalent circuit for EIS.

To further understand the effect of Sn/Be co-doping on the charge transfer kinetics of α–Fe_2_O_3_ photoanodes, EIS measurements were taken, as shown in Fig. 4a. Nyquist plots were obtained for all samples under standard illumination conditions, at an applied potential of 1.23 V_RHE_. In the Nyquist plots and the equivalent circuit, *R*_*S*_ is the series resistance, which includes mainly the sheet resistance of the FTO substrate and *R*_*CT1*_ and *CPE*_*1*_ elements that characterize the charge transfer resistance and the double layer capacitance at the FTO/α–Fe_2_O_3_ interface, respectively. *R*_*CT2*_ and *CPE*_*2*_ characterize the charge transport resistance and double layer capacitance of α–Fe_2_O_3_ and the α–Fe_2_O_3_/electrolyte interface, respectively^39^. As shown in the fitted results (Table S4), *R*_*CT1*_ decreased from 133.1 to 75.1 to 48.3 , while *R*_*CT2*_ decreased from 198.7–113.8 Ohms and finally to 87.3 Ohms for pristine, Sn-doped and co-doped α–Fe_2_O_3_ photoanodes, respectively. Both the Sn-doped and co-doped α–Fe_2_O_3_ photoanodes exhibited reduced electron transport resistance compared to pristine α–Fe_2_O_3_ photoanodes, consistent with the enhanced electrical conductivity upon Sn^4+^ mono-doping and Sn/Be co-doping. Sn/Be co-doped samples showed the lowest electron transport resistance and the highest capacitance values, in agreement with PEC and conductivity data. Hall Effect measurements were performed to determine the conductivity of pristine, Sn-doped and Sn/Be co-doped α–Fe_2_O_3_ photoanodes (Table S3). Conductivity values of co-doped α–Fe_2_O_3_ photoanodes were two times larger to those of pristine α–Fe_2_O_3_ photoanodes and slightly larger than those of Sn-doped α–Fe_2_O_3_ photoanodes, indicating that Be^2+^ co-doping along with Sn^4+^-dopant effectively increases conductivity. Figure 4(b) shows the Mott-Schottky plots of pristine and doped α–Fe_2_O_3_ photoanodes. Donor concentrations of photoanodes were calculated from the slopes of Mott-Schottky plots, yielding values of 1.21 × 10^19 ^cm^−3^, 7.64 × 10^19 ^cm^−3^ and 8.03 × 10^19 ^cm^−3^ for pristine, Sn-doped and Sn/Be co-doped α–Fe_2_O_3_ photoanodes, respectively. As discussed earlier, when Sn^4+^ is introduced into the α-Fe_2_O_3_ lattice, Fe^3+^ in the α-Fe_2_O_3_ lattice is replaced by Sn^4+^ donor dopants adjacent to Fe^3+^ sites. The Fe^3+^ ions are reduced to Fe^2+^ to preserve charge neutrality, and the newly formed Fe^2+^ sites result in improved electrical conductivity of α-Fe_2_O_3_ photoanodes. However, the two charge neutrality levels will have a residual mismatch, which can be easily overcome by co-doping with Be^2+^ dopants.

**Figure 5:**
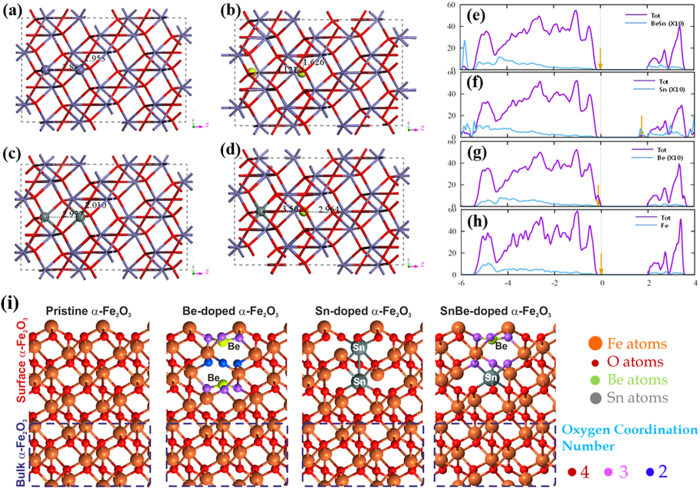
The optimized structure of (**a**) pristine, doped with (**b**) Be, (**c**) Sn, and (**d**) Be-Sn co-doped hexagonal α-Fe_2_O_3_. The GGA + U calculated PDOS with Gaussian broadening of 0.1 eV for **(e)** Be-Sn co-doped and doped with (**f**) Sn and (**g**) Be compared with (**h**) pure α-Fe_2_O_3_.(**i**) respective oxygen coordination states for pristine, Be-,Sn-, and Sn-Be-α-Fe_2_O_3_ photoanodes. The energy levels in different structures are aligned comparing the deep lying oxygen 2s orbital and the Fermi levels are shown with vertical (orange) arrows.

In order to analyze the doping effect on the electronic structure of α-Fe_2_O_3_, we carried out a series of first-principles calculations using density functional theory (DFT). We built four different systems, an undoped-, Be-, Sn- and Be/Sn-doped α-Fe_2_O_3_, with a dopant concentration corresponding to 3.3, 6.6 and 8.3 At%. These concentrations were selected to study diverse scenarios where either Sn or Be are predominant dopants, or when an equal concentration is found. We first focus on the systems doped at 3.3 At%. The optimized atomic structures and calculated density of states (DOS) of Be-, Sn-, and Sn/Be-doped α-Fe_2_O_3_ photoanodes are shown in Fig. 5(b–d), and compared to those of pure α-Fe_2_O_3_, Fig. 5(a). We observe that the undoped α-Fe_2_O_3_ exhibit an electronic band gap equal to 2.2 eV, in good agreement with the experimental value of 2.6 eV^40^. It is also well-known that the conduction band edge of α-Fe_2_O_3_ is highly localized, leading to a heavy electron-effective mass and, therefore, a very low electron conductivity^41^.

When Be is used to replace two Fe atoms, Be-doped α-Fe_2_O_3_, a sharp, partially-filled states appears on the valence band maximum (VBM) of α-Fe_2_O_3_, indicated by an arrow in Fig. 5g. The position of the peak with respect to the VBM is mainly determined by the O *2p* orbitals. As a result, the Fermi level for Be-doped α-Fe_2_O_3_ lies on the top of the valence band, thus inducing a *p*-type doping. Additionally, the Be^2+^ doping induce significant structural changes on the lattice structure of α-Fe_2_O_3_. From Fig. 5b we observe that the coordination number of Be^2+^ dopants decreases to 3 (6 for Fe^3+^ on undoped systems), via the breakage of several Be-O bonds along the Be-O-Be path. Therefore, the dangling bonds of oxygen atom form strongly localized impurity states around the Fermi level acting as scattering points for electron transport. The remaining Be-O bonds decrease in length up to 1.63 Å which indicates a strong interaction, and as a consequence, the Be-Be distance increase up to 4.12 Å, compared to 2.86 Å for the undoped case, leaving a small cavity between them, as seen in Fig. 5b. Thus, the Be dopant induces a clear red shift in the band gap transition by a 0.22 eV upshift in valence band edge of Be-doped α-Fe_2_O_3_ photoanodes compared to pristine α-Fe_2_O_3_ photoanodes. On the other hand, the introduction of Sn-dopant atoms apparently does not induce a significant lattice distortion on the atomic structure of α-Fe_2_O_3_, where the Sn atoms only move from the ideal position by 0.06 Å, see Fig. 5c, and the distance between two Sn atoms is 2.98 Å, just 0.12 Å larger than the undoped case. However, Sn-doping actually induce a larger strain, accompanied with a very shallow donor level below the conduction band minimum (CBM), as indicated by the DOS in Fig. 5f, inducing an *n*-type doping, contrary to the Be-doped system. The band gap of Sn-doped α-Fe_2_O_3_ photoanodes shrinks by ~0.40 eV compared to pristine α-Fe_2_O_3_ photoanodes, and the Fermi level moves to a higher energy level, eventually laying on the conduction band edge. In addition, the concentration of free electrons is increased due to the new donor level^42^,^43^, resulting on enhanced photoactivity when compared with Be-doped and pristine α-Fe_2_O_3_. Despite of the increased charge carrier density, the newly created impurity levels might still act as recombination centers for electron-hole pairs, therefore, removing those localized states is a key step towards a photoanode with improved PEC performance. In this case, by introducing simultaneously Sn/Be onto α-Fe_2_O_3_ photoanodes, the structural deformation and lattice strain is less pronounced than the Be- or Sn-doped α-Fe_2_O_3_ cases, as shown in Fig. 5d. From Fig. 5e, we observe that the electronic structure is very similar to that of pristine α-Fe_2_O_3_ photoanodes, similar results are obtained for dopant concentration of 6.6 At% (See Fig. S7). However, this is not the case when the Sn and Be concentrations are dissimilar, as explained below. Interestingly, both localized impurity peaks at VBM observed in Be-doping, and the shallow *n*-type defect state beneath the CBM observed in Sn-doping are compensated on the Sn/Be co-doped α-Fe_2_O_3_ case, thus minimizing the presence of recombination centers. Additionally, the CBM of Sn/Be co-doped α-Fe_2_O_3_ exhibit less localized features suggesting a smaller effective electron mass, and hence, improved electron conductivity when compared with the pristine α-Fe_2_O_3_, similar effects has been observed for Ti/N co-doped hematite^44^. As a result, the charge carrier density is greatly improved, and due to fewer recombination sites, the Sn/Be co-doped α-Fe_2_O will exhibit enhanced photoactivity, in agreement with our experimental observations. On the other hand, doped α-Fe_2_O_3_ systems with dissimilar concentration of Sn and Be exhibit an intermediate behaviour when compared with a single doped (Sn or Be) and 1:1-Sn/Be co-doped samples. For example, we observed that a co-doped α-Fe_2_O_3_ with 5 and 3 At% of Sn and Be, respectively, (5Sn/3.3Be-co-doped Fe_2_O_3_) exhibit *n*-type characteristics with a reduced band gap (1.85 eV) when compared with non-doped α-Fe_2_O_3_. The excess of Sn dopant generates intermediate peaks in the band gap (See Fig. S7b), however, the density of localized states is significantly lower than those single-doped systems with just Sn or Be dopants (See Fig. S7c).

Bader charge analysis^45^ shows that Fe atom lose ~1.7 electrons in pure Fe_2_O_3_, whereas the charge states of Sn and Be dopants were calculated to be Sn^+2.2^ and Be^+1.4^ in Sn-Be co-doped α-Fe_2_O_3_ photoanodes, respectively. From the above results, Be dopants substituting in Fe sites should be regarded as the *p*-type dopant, whereas Sn should be regarded as the *n*-type dopant, in agreement with previous experimental reports^10^,^43^. The carrier mobility is significantly affected on Be-doped α-Fe_2_O_3_ photoanodes due to the localized nature of dopant-induced states at the top of the valence band. Similarly, in the case of Sn-doping, the Coulomb scattering induced by charged defect (donor) sites results in reduced carrier mobility.

Finally, by investigating the formation energy, the relative difficulty to incorporate Sn or Be dopants into the α-Fe_2_O_3_ lattice was evaluated. The formation energy of Sn/Be co-doped α-Fe_2_O_3_ (0.44 eV) is considerably smaller than that of Be- (2.47 eV) or Sn- (4.26 eV) doped α-Fe_2_O_3_ photoanodes. Since the optimized structures are obtained by relaxing only the atomic coordinates (keeping the crystal structure unchanged), large formation energies imply not only low dopability, but also reduced structural stability. Because of the large formation energy, Be or Sn-mono dopant induces large structural distortion and breaks the crystallinity of α-Fe_2_O_3_, which results in low electrical conductivity in spite of high carrier concentration from dopants. In addition, we also performed geometric optimization with a variable cell scheme to determine the change in lattice parameter, and hence the induced strain. The magnitudes of the induced strain measured along the (220) crystal plane are ~1% for both Sn-, and Be- doped systems, but just ~0.2% for Sn/Be-doped, see Fig. S8, these results fits remarkably well with the synchrotron XRD profiles as explained below.

**Figure 6:**
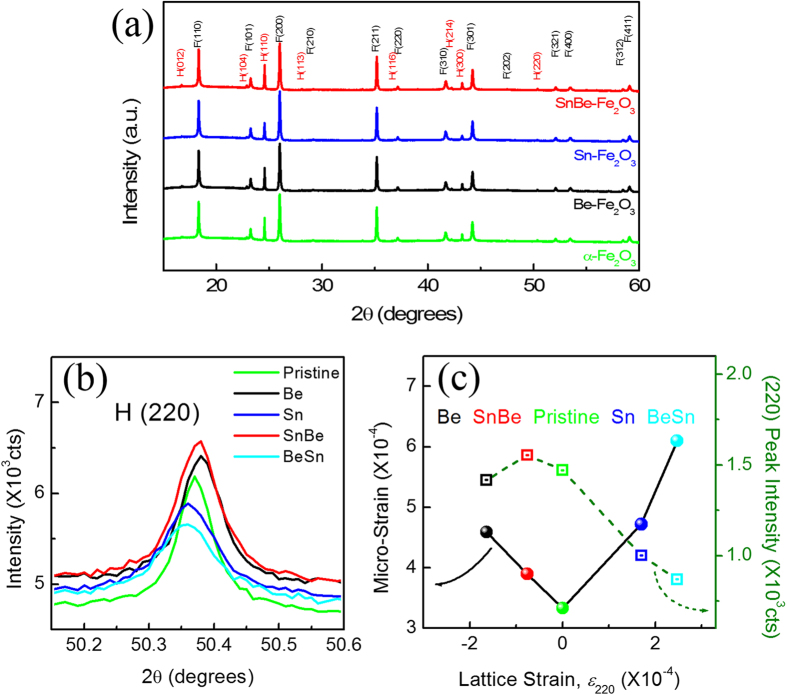
(**a**) Full XRD profiles and (**b**) hematite (220) diffraction profiles of pristine, Be-doped, Sn-doped, and SnBe co-doped photoanodes sintered at 800 °C. (**c**) The variations of micro strain (black line) and (220) diffraction intensity (green dashed line) as function of lattice strain of the photoanodes.

Figure 6(a) shows the synchrotron XRD profiles of the pristine, Be-doped, Sn-doped, and Sn/Be co-doped α–Fe_2_O_3_ photoanodes. With the exception of FTO substrate peaks (denoted as ‘F’, JCPDS 41–1445), all peaks can be indexed to the α–Fe_2_O_3_ phase (denoted as ‘H’, JCPDS 33–0664). Aside from those of α–Fe_2_O_3_, no diffraction peaks of Sn, Be or other impurity phases were observed, indicating that the doping does not significantly disturb the crystal structure of α–Fe_2_O_3_. The pristine and doped α–Fe_2_O_3_ photoanodes displayed similar XRD patterns, with a predominant diffraction peak at the (110) plane as well as the (220) plane^10^,^31^. In order to evaluate the effect of doping on the average crystallite size and micro strain, the Williamson-Hall method was utilized by the equation (1) 46.


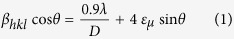


where *β* is the integral breadth of the peak from the (*hkl*) plane, *θ* is the Bragg angle, *D* is the average crystallite size, and *ε*_*μ*_ is the micro-strain. These parameters are summarized in Table 1. Interestingly, Sn or Be single-dopant doping increases the micro strain on the photoanodes by more than 35%, however, it reduced by Sn/Be co-doping effect, in good agreement with our theoretical observations. Otherwise, Be/Sn co-doping sequence results in the highest micro-strain. Meanwhile, a clear angle shift of the hematite (220) peak was observed, as shown in Fig. 6b. Both the amount and direction of angle shift were dependent on doping sequence. As a result, the lattice strain, expressed by 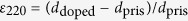, where *d*_doped_ (*d*_pris_) is *d*-spacing of the hematite (220) plane for the pristine sample, was obtained and also summarized in Table 1. The micro-strain versus lattice strain was depicted in Fig. 6c. Both strains were minimized in the Sn/Be co-doped photoanode sample, as indicated by our DFT calculations. However, the Be/Sn co-doping sequence accumulated the highest lattice strain and micro-strain, most probable because Be will first occupy the most preferable and large sites, resulting in larger number of under-coordinated O atoms, and also leaving smaller or unsuitable doping sites for Sn in Fig. 5i. Under-coordinated O atoms induced as a result of Be/Sn co-doping sequence might act as potential recombination centers and hence contributes to undesired electron recombination. On the other hand, when Sn is introduced first, Sn will occupy those larger sites reducing the under-coordinated atoms, and leaving the smaller ones to Be, resulting in a more efficient doping. Thus the sequence of *ex-situ* co-doping is very important. In addition, Sn/Be co-doped photoanodes showed the highest (220) peak intensity. Otherwise, Be/Sn co-doped photoanodes exhibited the lowest intensity, indicating a lower crystalline order might be related a larger lattice distortion.

**Figure 7:**
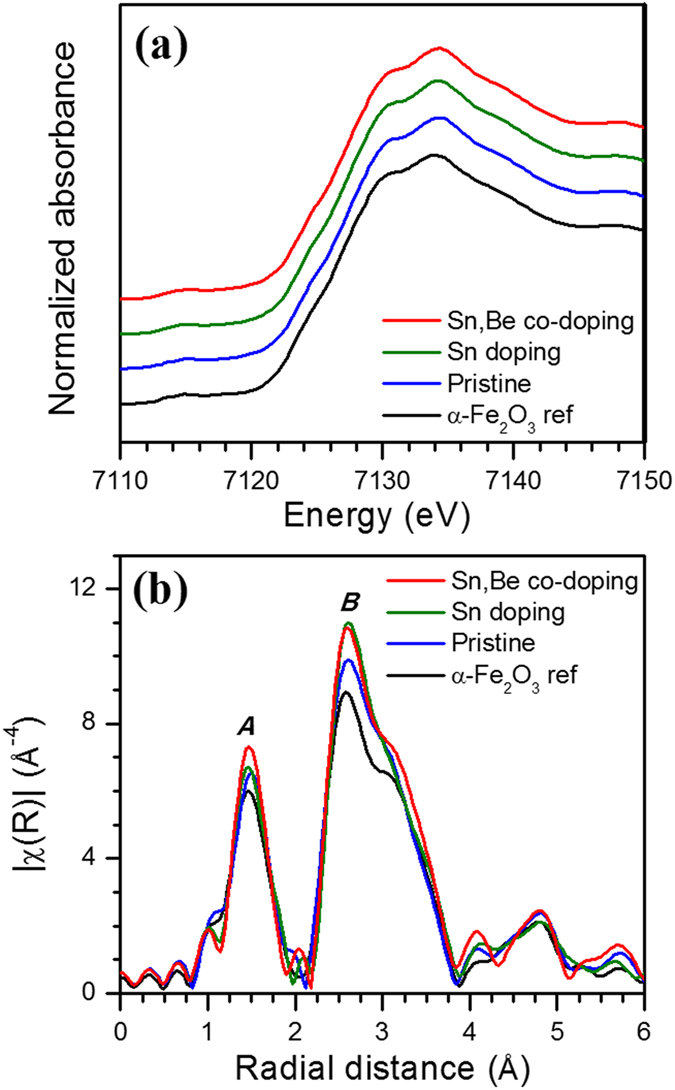
(**a**) XANES spectra and (**b**) k3-weighted Fourier transforms of EXAFS functions for Fe K-edges of pristine, Sn-doped, and co-doped α-Fe_2_O_3_ photoanodes sintered at 800 °C. α-Fe_2_O_3_ reference in powder is included for comparison.

XAFS is an element-specific and bulk-local structure-determining probe. Figure 7 displays X-absorption near-edge structure (XANES) spectra and Fourier-transformed spectra of extended X-ray absorption fine structure (EXAFS) functions for Fe K-edges of doped α-Fe_2_O_3_ photoanodes. The XANES spectra for the samples were exactly the same as those of reference α-Fe_2_O_3_. The pre-edge peak denoting a quadrupole transition of *1s* → *3d* were observed at 7,115 eV, and the absorption rising feature and energy positions were the same. Doped α-Fe_2_O_3_ photoanodes exhibited two peaks in the Fourier transforms of EXAFS functions; the first peak *A* at 0.8–2.0 Å is due to the nearest Fe-L (L=O or Be) bond, while the second peak *B* at 2.1–3.9 Å is the contribution from Fe-M (M=Fe or Sn) and Fe-O bonds at a greater distance. Compared with reference α-Fe_2_O_3_ in powder, the samples exhibited increased intensities in both peaks, indicating enhanced orderings of the respective bonds in the films on substrates. It is a noteworthy observation that Be mono-doped and Sn/Be co-doped photoanodes had the highest intensity for the peak *A*. In the case of pristine samples, the Sn diffused from the FTO substrates had a detrimental effect on Fe-O bonds. While the Fe-O bond for Sn-doped sample was affected by both Sn diffused from FTO and dopant on calcination, stabilization of Be^2+^ dopant in Sn^4+^ in the co-doped sample would have had a positive influence on the nearest Fe-O bond, resulting in improved bond ordering. The enhancing effect in the structural ordering by Be doping is also confirmed as the highest intensity for Be mono-doped Fe_2_O_3_. Beryllium is doped into the position of oxygen in hematite lattice and thus, the scattering on Fe atom from neighboring Be will be much weaker than that from neighboring O unless the structural ordering is considered. On the other hand, for the peak *B*, both co-doped and mono-doped samples had increased intensities compared with pristine α-Fe_2_O_3_. This effect is not due to enhanced bond ordering, but rather to strong backscattering from Sn in those samples. When both Fe and Sn (types of metal scatterers) are present in the samples, Sn overwhelms the scattering from the lighter Fe atom^47^.

Pristine α-Fe_2_O_3_ photoanodes have low electron conductivity due to heavier electron-effective masses. When α-Fe_2_O_3_ photoanodes were doped with Sn, the Sn^4+^ dopants introduced a shallow donor level below the conduction band minimum of α-Fe_2_O_3_, which enhanced electron conductivity and PEC properties of Sn-doped α-Fe_2_O_3_ photoanodes. However, Sn^4+^ dopants induced a distortion in α-Fe_2_O_3_ structure, which was further confirmed with an increase in micro-strain and lower bond-ordering. The observed defects can be effectively overcome by co-doping with Be^2+^. The shallow, *n*-type defects introduced by Sn^4+^ doping can be overcome with Sn/Be co-doping, resulting in further enhancement of electrical conductivity. This is done by improving charge carrier density while mobility remains unchanged, leading to improved bond-ordering, reduced micro-strain and further enhancement in photocurrent (1.7 mA/cm^2^) with minimal transport resistance for Sn-Be co-doped α-Fe_2_O_3_ photoanodes.

## Conclusion

In summary, α-Fe_2_O_3_ photoanodes sequentially co-doped with Sn^4+^ and Be^2+^ were investigated as means for efficient PEC water splitting. The photoactivity of α-Fe_2_O_3_ was remarkably improved by Sn^4+^ and Be^2+^ co-doping. Maximum photocurrent density was exhibited by the Sn(4%)-Be(6%) co-doped α–Fe_2_O_3_ photoanode (1.7 mA/cm^2^), with a photocurrent density two times larger than pristine α–Fe_2_O_3_ photoanodes. Both the Sn-doped and Sn/Be co-doped α–Fe_2_O_3_ photoanodes exhibited lower electron transport resistances compared to undoped samples, consistent with the enhanced electrical conductivity upon Sn^4+^ mono-doping and Sn-Be co-doping. From DFT calculations, the localized impurity peak at valance band maximum in Be doping and the shallow *n*-type defect state beneath the conduction band minimum in Sn doping are effectively balanced by Sn/Be co-doping. Sn^4+^ dopants introduced undesired band shifts and increased micro-strain in α-Fe_2_O_3_ photoanodes. This issue was resolved by employing Be^2+^ as a co-dopant, and ultimately confirmed with DFT, EXAFS and synchrotron XRD studies.

### Experimental Section

α-Fe_2_O_3_ nanorods on FTO glass were prepared using a simple hydrothermal method, as reported by Vayssieres *et al.*^48^. In typical fashion, a piece of cleaned FTO glass was placed within a vial containing a solution consisting of 0.4 g FeCl_3_•6H_2_O and 0.85 g NaNO_3_ at pH 1.5 (adjusted by HCl)^29^. The hydrothermal reaction was conducted at 100 °C for 6 h. After cooling to room temperature, the FTO glass was rinsed several times with distilled water and dried at 60 °C. Annealing at 550 °C for 4 h was carried out for the phase transition from β–FeOOH to pure α–Fe_2_O_3_ 49. The *ex-situ* Sn doping was carried out using a simple dipping method to treat the α-Fe_2_O_3_ photoanodes with a Sn precursor solution^30^. A similar procedure was followed for Be doping using BeSO_4_ as a Be precursor. Different concentrations of SnCl_4_ dissolved in ethanol and BeSO_4_ dissolved in deionized water were used to prepare the Sn and Be precursor solutions, respectively. After dipping, the photoanodes were allowed to dry in air at room temperature. The surface-treated samples were then subjected to high temperature sintering. This high temperature sintering (800 °C for 10 min) is believed to be important for activating the α-Fe_2_O_3_ photoanodes by enhancing electron transfer between α-Fe_2_O_3_ and conductive substrates^50^,^51^.

X-ray diffraction (XRD) patterns of all samples were collected using an X-ray diffractometer (Rigaku RINT 2500) with CuKα radiation. The surface morphology of the samples was analyzed using field emission scanning electron microscopy (FESEM, JEOL JSM 700F). X-ray absorption fine structure (XAFS) experiments were carried out on the 7D beamline of the Pohang Accelerator Laboratory (PLS-II, 3.0GeV). Synchrotron radiation was monochromatized using a Si (111) double crystal monochromator. At room temperature, the spectra for the Fe K-edge (*E*_*0*_ = 7112 eV) were taken in fluorescence mode. The incident beam was detuned by 30% for the Fe K-edge in order to minimize contamination of higher harmonics. The intensity of the incident beam was monitored using a He-filled IC SPEC ionization chamber. The fluorescence signal from the sample was measured with a passivated implanted planar silicon (PIPS) detector. During the measurements, helium was continuously pumped into the sample chamber to minimize fluorescence signals for spectra with elevated signal-to-noise ratios. AHENA in the IFEFFIT suite of software programs was used to analyze the data for the local-structure study of Fe in doped α-Fe_2_O_3_ photoanodes^52^. Pristine, Be-doped, Sn-doped, and co-doped α–Fe_2_O_3_ photoanodes were investigated by XRD measurement with the 5A beamline of the Pohang Light Source II (PLS-II) in Korea. The XRD data was obtained with conventional theta/two-theta scans, at a wavelength of 0.1072 nm. All photoelectrochemical measurements were carried out in 1 M NaOH (pH = 13.8) using a potentiostat (Ivium CompactStat) with a Pt coil as the counter electrode and Ag/AgCl as the reference electrode. Photocurrent-potential (*J-V*) curves were swept at 50 mVs^−1^ from −0.7 to +0.7 V compared to Ag/AgCl. To measure incident photon-to-current conversion efficiencies (IPCE), a 300 W Xe lamp (Newport, 6258) was coupled to a grating monochromator (Newport, 74125) operating in the wavelength range of 330–600 nm, and incident light intensity was measured with a UV silicon detector (Newport, 71675). The photoelectrode was biased at +0.6 or +1.0 V (compared to Ag/AgCl) during all IPCE measurements. Impedance spectroscopy measurements were performed using an impedance analyzer (Ivumstat). Impedance spectra were measured over a frequency range of 1 × 10^−2^ to 3 × 10^6 ^Hz at 25 °C under open circuit conditions, with amplitude of 10 mV and under a bias illumination of 100 mWcm^−2^. For ICP analysis, 39.34 mL of conc. HCl (37%) solution was taken in a 100 mL Teflon beaker. 10 mL of conc. HNO_3_ (70%) solution was slowly added to conc.HCl solution. Photoanodes were dipped in aqua regia solution up to film dissolution. In each condition 10 samples were dissolved with aqua regia solution. Atomic ratio of elements in as-prepared hematite samples were measured by inductively coupled plasma mass spectrometry (ICP-MS; Agilent 2500a, Santa Clara, CA, USA).

### Computational Details

Ab initio calculations were performed within the framework of the density functional theory (DFT)^53^ using the generalized gradient approximation and the model of Perdew, Burke and Ernzerhof^54^ as the exchange-correlation term. The electronic structure was solved using Vanderbilt ultrasoft pseudopotentials^55^. An kinetic energy cutoff for wave functions was set to 40 Ry and 360 Ry for the charge density. A Marzari-Vanderbilt^56^ smearing of 0.01 Ry was used to aid convergence. The integration of the Brillouin zone was carried out using 3 × 3 × 3 Monkhorst-Pack grid^57^. The DFT computations were performed using the Quantum Espresso (QE) code^58^ .We also compared our results with the VASP code^59^,^60^ using a cut-off energy of 500 eV, the rest of the variables were kept similar to those used in QE. In order to take into account the strong on-site Coulomb interaction present in *d* orbitals of Fe, we adopted the LSDA+U formalism as described by Dudarev, *et al.*^61^. An effective Hubbard correction term, U_eff_, of 4.5 eV was used to properly describe the electronic structure of α–Fe_2_O_3_ 62,63. A super cell oriented towards the [220] crystal direction containing 60 atoms was used as a model, where the doping was performed on two contiguous Fe atoms with different spin orientations, and thus maintaining an antiferromagnetic configuration^64^. These two contiguous Fe atoms were replaced by Be, Sn, and a co-doped Be/Sn system was also investigated. Finally, the ion positions were geometrically optimized by conjugate gradient minimization.

## Additional Information

**How to cite this article**: Annamalai, A. *et al.* Sn/Be Sequentially co-doped Hematite Photoanodes for Enhanced Photoelectrochemical Water Oxidation: Effect of Be^2+^ as co-dopant. *Sci. Rep.* **6**, 23183; doi: 10.1038/srep23183 (2016).

## Supplementary Material

Supplementary Information

## Figures and Tables

**Table 1:** Parameters of crystallite size (*D*) and micro-strain (*ε*) determined from Williamson-Hall plot.

Photoanode	Crystallite size *D*(nm)	Micro-strain *ε*_*μ*_ (×10^−4^)	*d*_220_spacing (nm)	Lattice strain *ε*_*μ220*_ (×10^−4^)
*α*-Fe_2_O_3_	103.0	3.33	0.12591	−1.64
Be-Fe_2_O_3_	104.6	4.59	0.12589	−0.76
Sn-Fe_2_O_3_	78.5	4.72	0.12593	0.00
Sn-Be-Fe_2_O_3_	89.1	3.90	0.12590	1.70
Be-Sn-Fe_2_O_3_	75.1	6.10	0.12594	2.47

Lattice plane distance (*d*_220_) and lattice strain (*e*_220_) were obtained from peak position in Fig. 5(b).
