# Supplementary material for: Sn/Be Sequentially co-doped Hematite Photoanodes for Enhanced Photoelectrochemical Water Oxidation: Effect of Be2+ as co-dopant
Source: Sci Rep. 2016 Mar 23;6:23183. doi: 10.1038/srep23183 (PMC4804299; doi:10.1038/srep23183)
Supplement: Supplementary Information [file srep23183-s1.doc]

**Supporting Information**

Sn/Be Sequentially Co-doped Hematite Photoanodes for Enhanced Photoelectrochemical Water Oxidation: Effect of Be^2+^ as a co-dopant

Alagappan Annamalai**^1^**, Hyun Hwi Lee**^2^**, Sun Hee Choi**^2^**, Su Yong Lee**^2^**, Eduardo Gracia-Espino^3^, Arunprabaharan Subramanian^1^, Jaedeuk Park^4^, Ki-jeong Kong**^4*^** and Jum Suk Jang**^*1^**

**^1^**Division of Biotechnology, Advanced Institute of Environmental and Bioscience, College of Environmental and Bioresource Sciences, Chonbuk National University, Iksan 570-752, Republic of Korea.

***^2^****Pohang Accelerator Laboratory, POSTECH, Pohang 790-784, Republic of Korea.*

*^3^Department of Physics, Umeå University, Umeå, SE-901 87, Sweden.*

***^4^****Center for Chemical Safety and Security, Korea Research Institute of Chemical Technology (KRICT) Daejeon, 305-343, Republic of Korea.*

***** Corresponding author. E-mail address:

[kong@krict.re.kr](mailto:kong@krict.re.kr) (KJK) and [jangjs75@jbnu.ac.kr](mailto:jangjs75@jbnu.ac.kr) (JSJ)


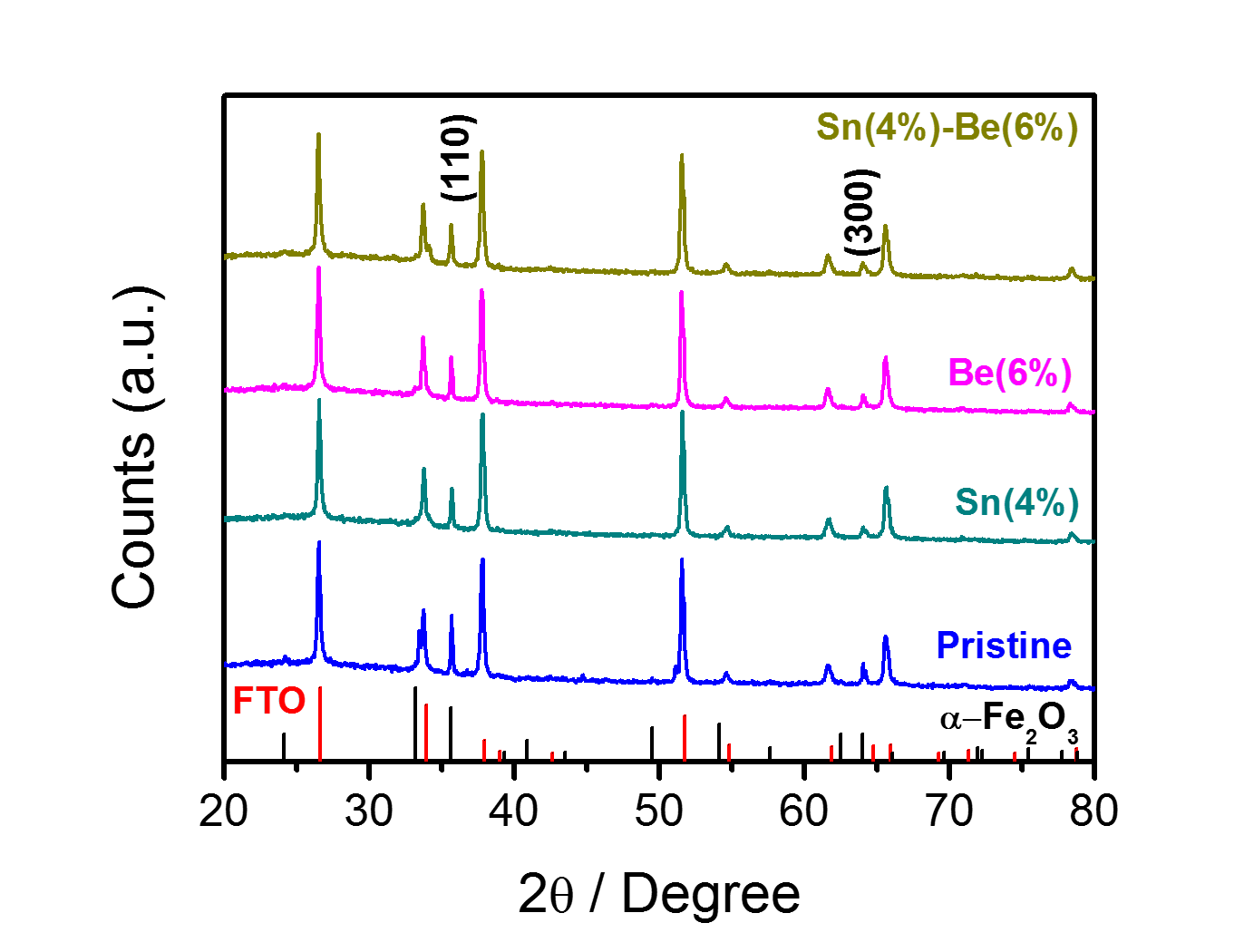


**Figure S1.** X-ray diffraction patterns of pristine, Sn-doped, be-doped and co-doped α–Fe_2_O_3_ photoanodes sintered at 800 °C.

**
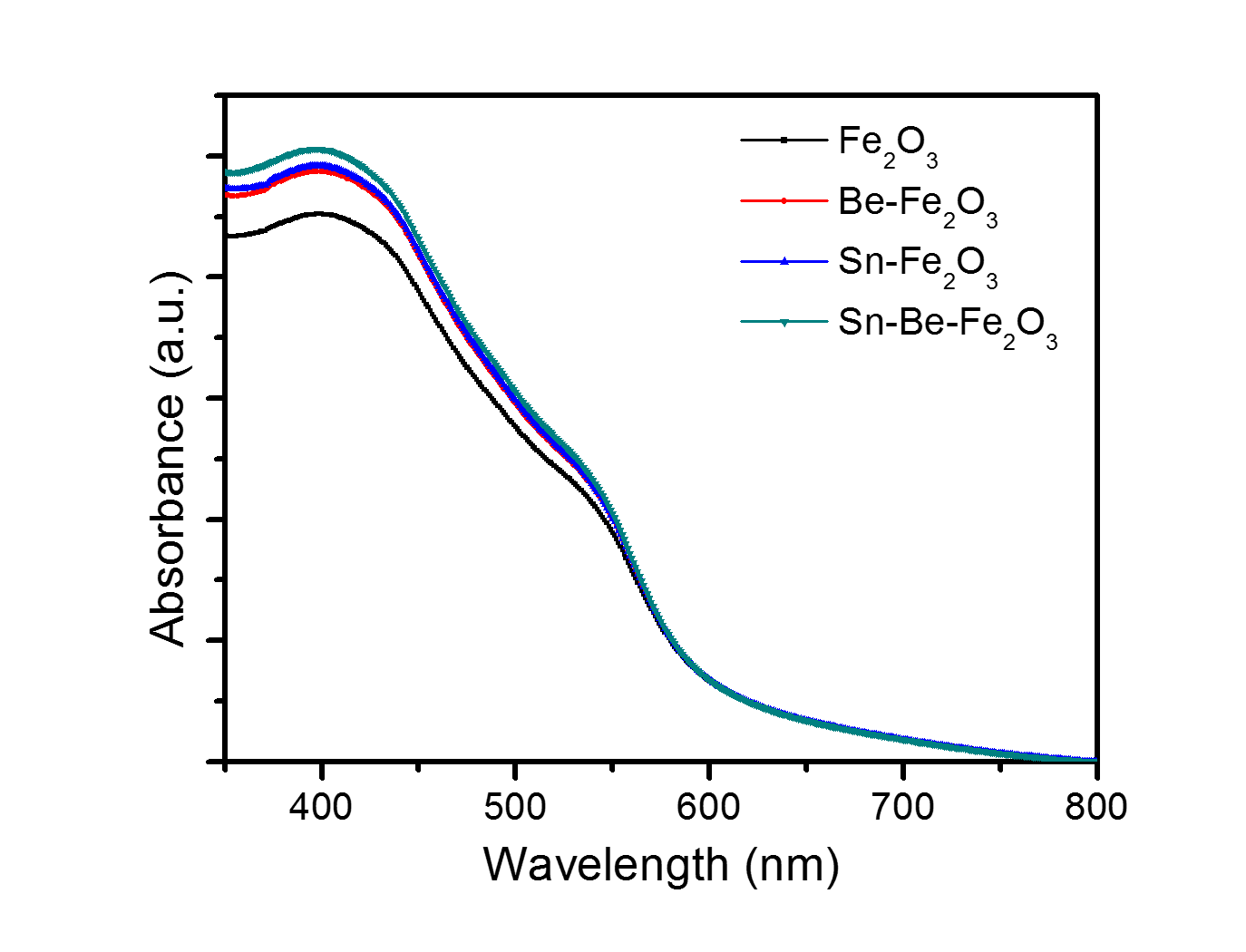
**

**Figure S2.** Optical property of pristine, Be-doped, Sn-doped, co-doped α–Fe_2_O_3_ photoanodes sintered at 800°C, which shows the absorption coefficient as a function of wavelength. The band gap energy of α–Fe_2_O_3_ photoanodes namely pristine, Sn-doped, Be doped and Sn-Be co-doped α–Fe_2_O_3_ photoanodes are determined as 2.12 eV, 2.14 eV, 2.13 eV and 2.12 eV respectively, which are in accordance with the typical values of α–Fe_2_O_3_ photoanodes. It could be deduced that doping has not produced intermediate band levels within the bandgap of α–Fe_2_O_3_ since doping does not change the bandgap of α–Fe_2_O_3_ photoanodes.


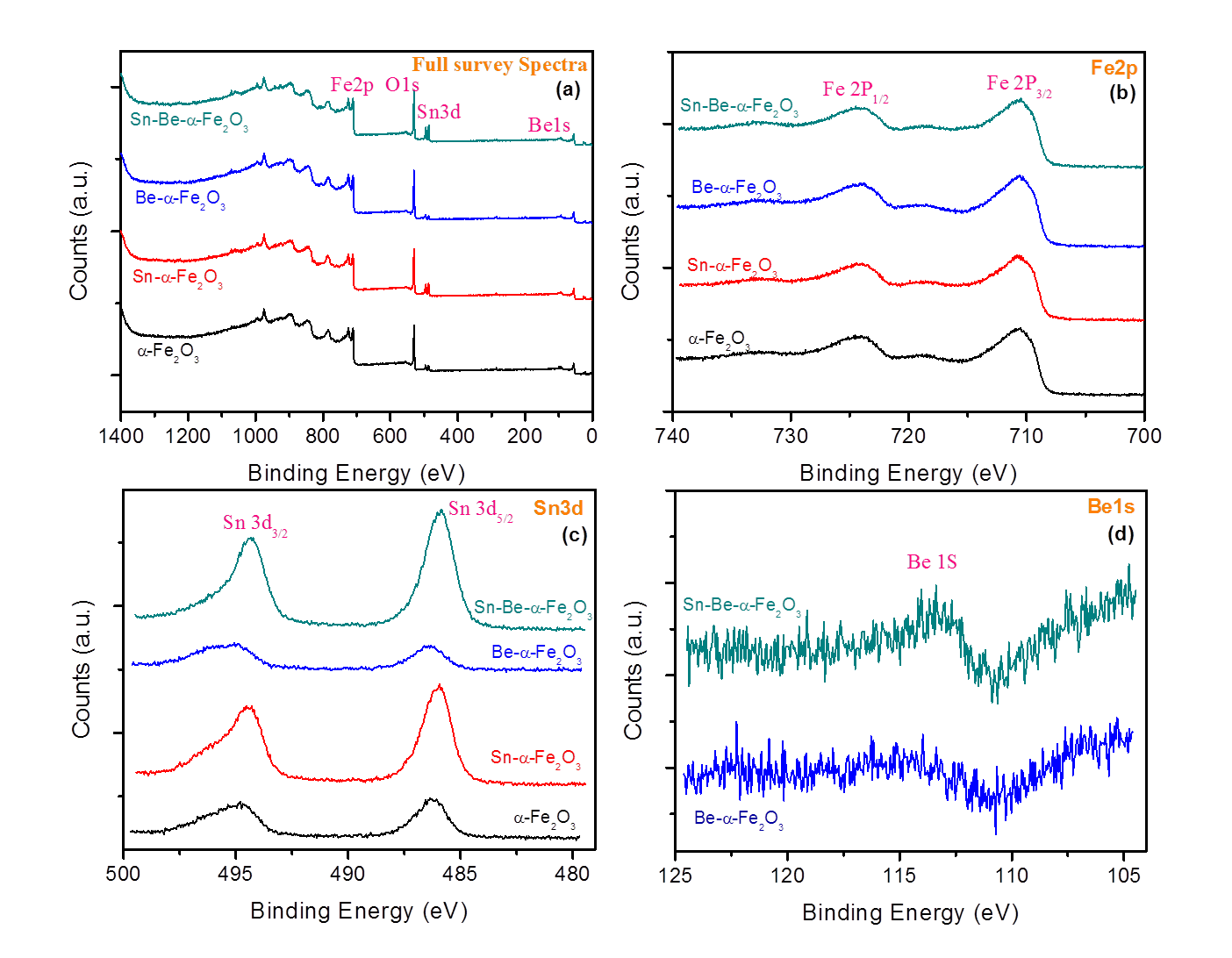
**Figure S3.** X-ray photoelectron spectra of (a) full survey spectra, (b) Fe 2p, (c) Sn 3d and (d) Be 1s recorded from pristine, Sn^4+^, Be^2+^ and Sn/Be co-doped –Fe_2_O_3_ photoanodes sintered at 800 °C.

**
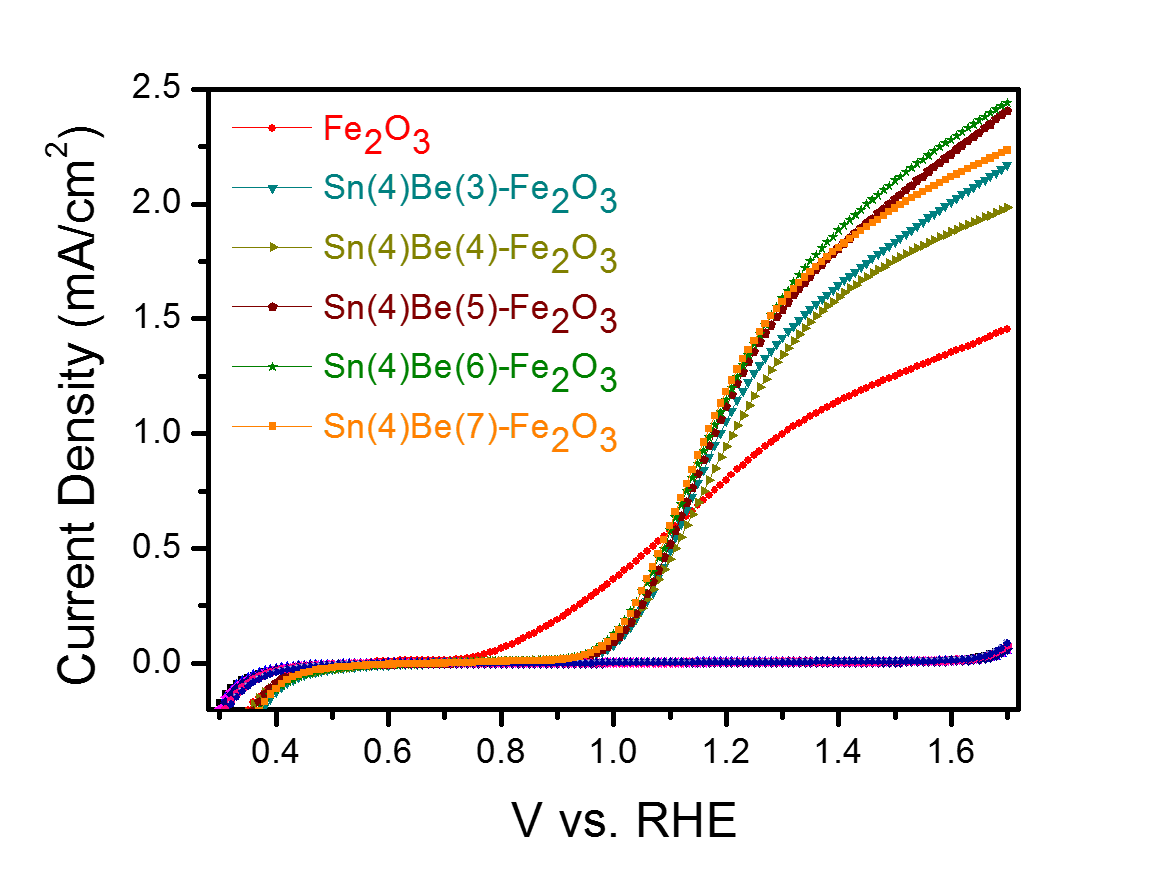
**

**Figure S4.** Photocurrent-potential (*J-V*) curves for PEC water oxidation reaction of Pristine and co-doped α–Fe_2_O_3_ photoanodes annealed at 800°C under standard illumination conditions. Photocurrent was sensitive to the concentration of Be-dopant. There was a decrease in photocurrent increasing the Be-dopant concentration more than 6%. When Be^2+^ dopants were introduced as co-dopants, they tend to recover the shift in conduction band and reduce the micro-strain caused by Sn^4+^ mono-dopants. However, when the concentration of Be^2+^ dopants is above the optimum concentration, they tend to overshadow the improved charge transport properties by Sn^4+^-dopants as Be^2+^ dopants does not contribute the electronic properties (charge carrier concentration).


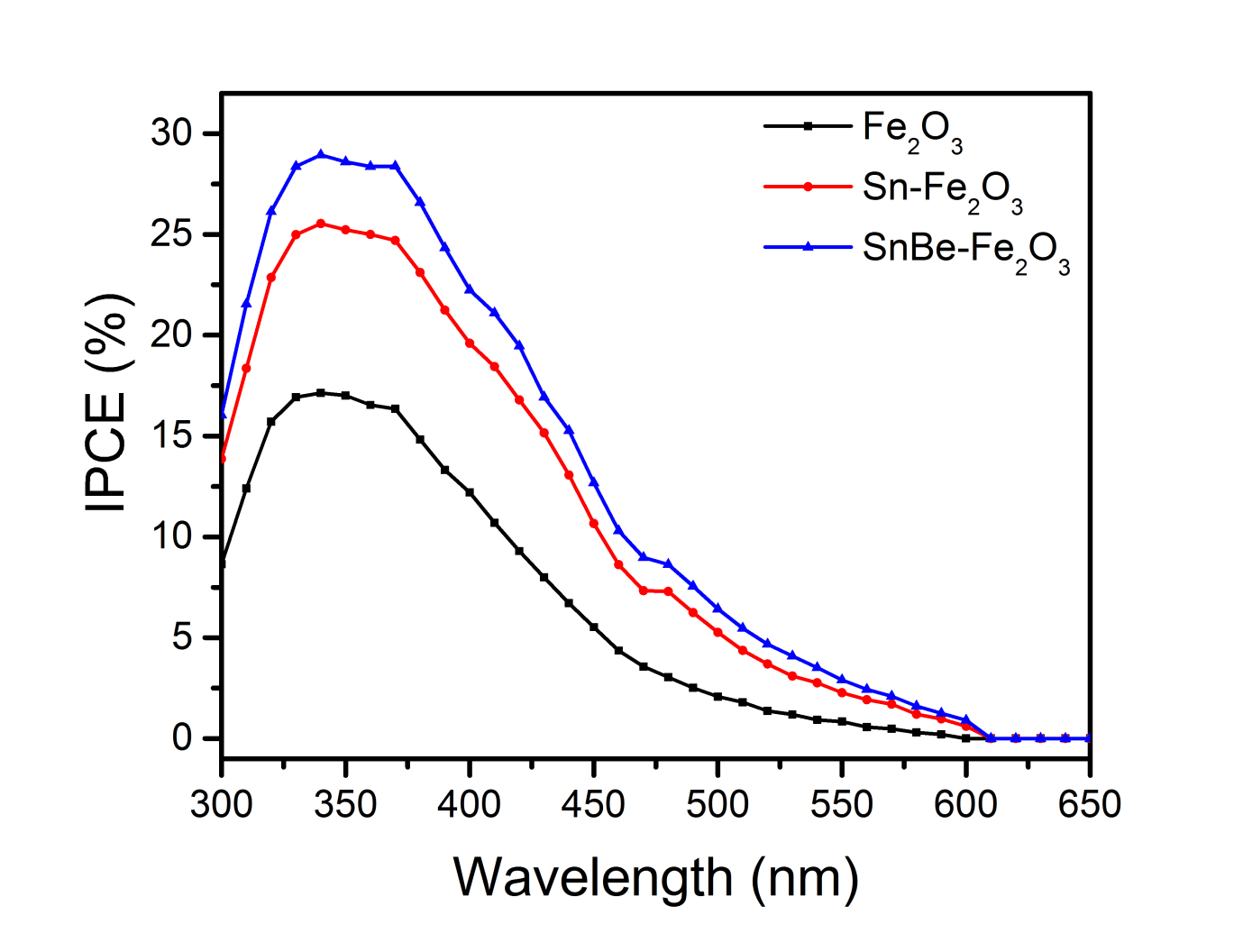
**Figure S5.** Incident photon-to-current-efficiency (IPCE) spectra for PEC water oxidation reaction with pristine, Sn-doped and co-doped α–Fe_2_O_3_ photoanodes measured at 1.23 V_RHE_, under standard illumination conditions.


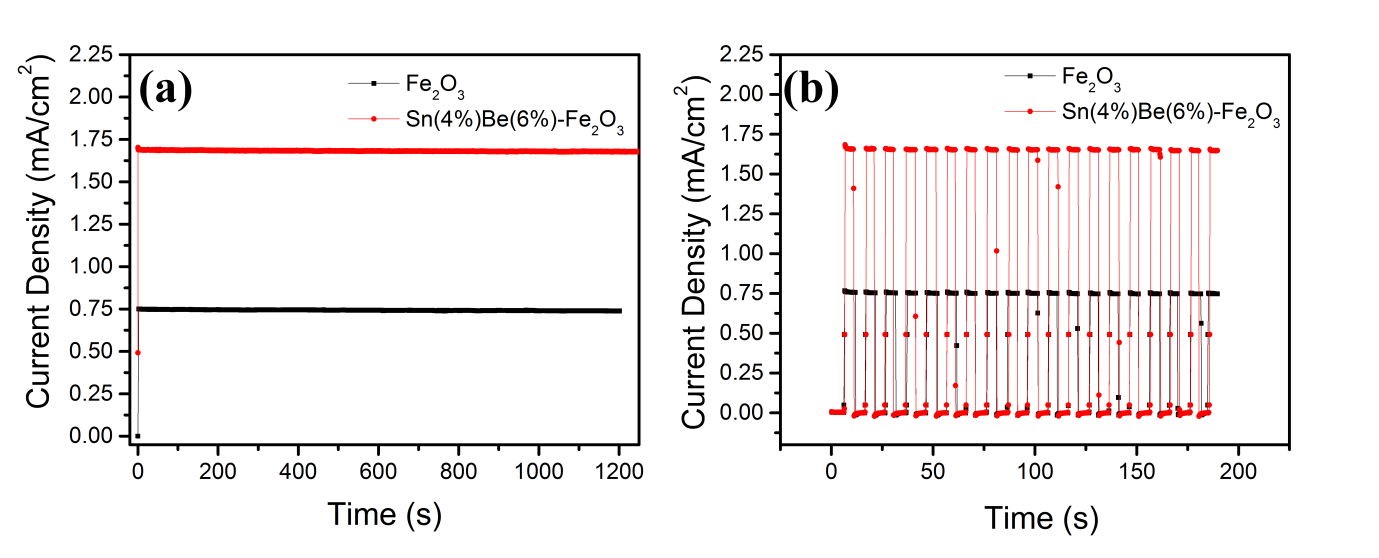


**Figure S6.** Photochemical stability and (b) photochemical response at 1.4 V_RHE_ under standard illumination conditions for pristine α–Fe_2_O_3_ photoanodes and Sn and Be Co-doped α–Fe_2_O_3_ photoanodes. The co-doped α–Fe_2_O_3_ photoanodes showed relatively longer electron lifetime, contributing to its high PEC performances, in good agreement with the above results. The co-doped α–Fe_2_O_3_ stability was tested at 1.4 V_RHE_ under standard illumination conditions for 1200 seconds. There is no observable degradation, indicating excellent chemical and structural stability of the co-doped α–Fe_2_O_3_ photoanodes for long-term PEC conversion. We further performed the photostability tests, in comparison to pristine α–Fe_2_O_3_ photoanodes, the co-doped α–Fe_2_O_3_ photoanodes have excellent photostability.


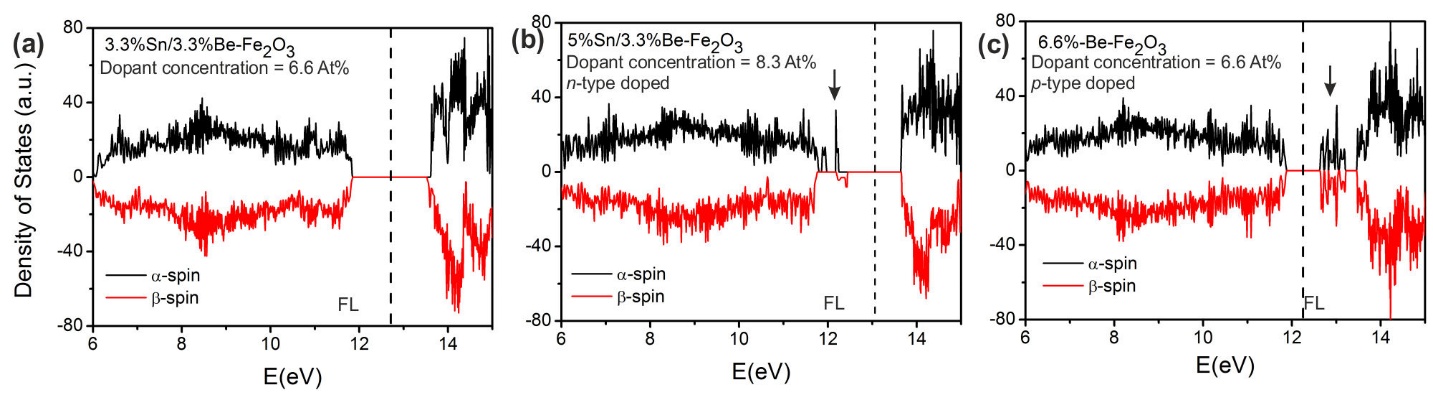


**Figure S7.** Spin polarized density of states of (a) Sn/Be-Fe_2_O_3_ with equal doping concentration of both Sn (3.3 At%) and Be (3.3 At%), where no intermediates states in the band gap are found. (b) Sn/Be-Fe_2_O_3_ with an excess of Sn (5 At% Sn, 3.3 At% Be), where some localized states appears below the Fermi level (FL). This system exhibit n-type doped characteristics. (c) Be-Fe_2_O_3_ with 6.6 At% of Be, where larger number of localized states (possible recombination sites) appears above the FL, the system exhibit a p-type doped characteristics.


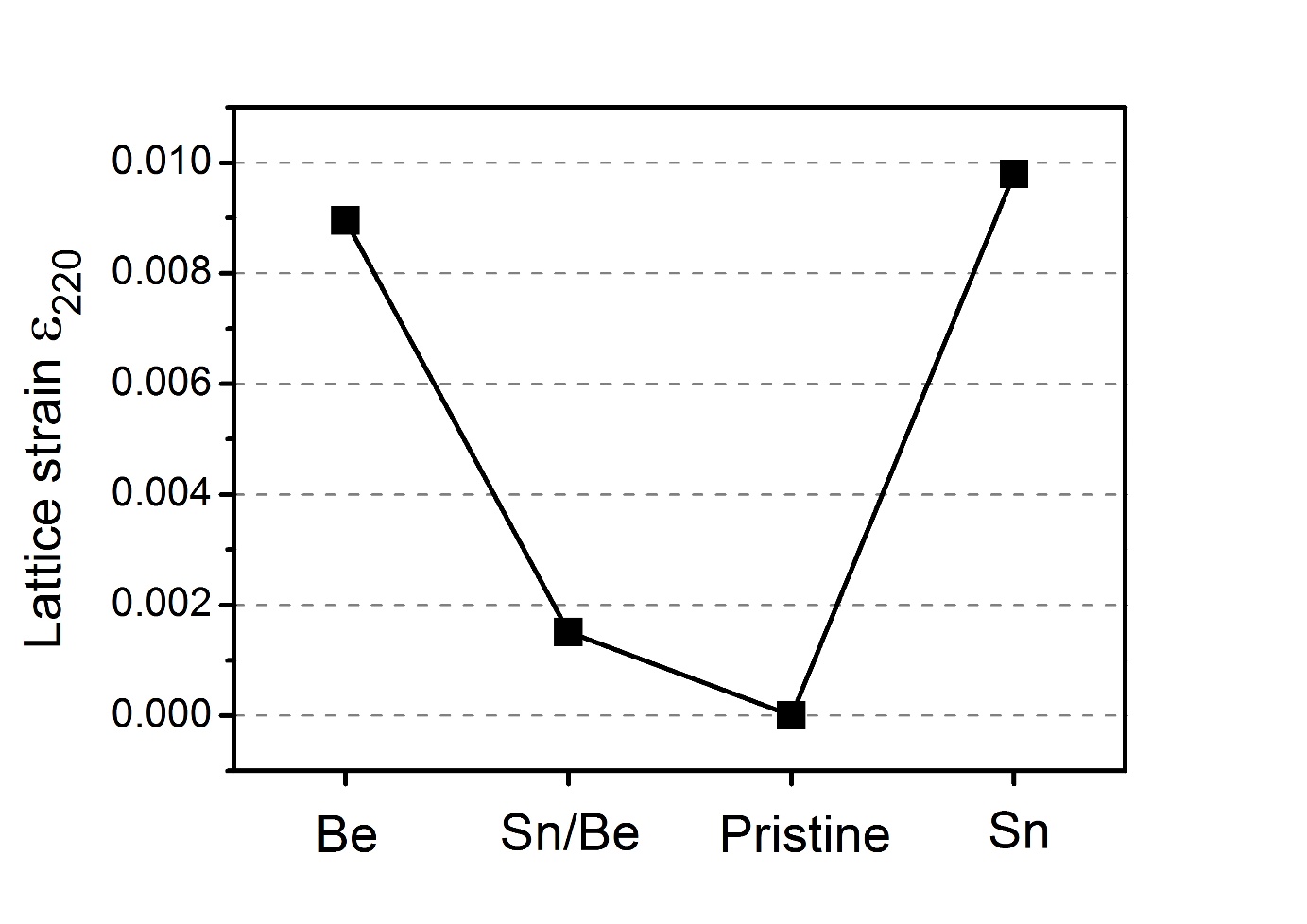


**Figure S8.** Absolute value of the lattice strain generated by the introduction of Be, Sn, and Sn/Be into the α-Fe_2_O_3_. The lattice strain is calculated along the (220) crystal plane.

**Table S1.** Atomic ratio of elements in as-prepared hematite samples was obtained by ICP-MS.

| **Samples** | **Sn/Fe atomic ratio** | **Be/Fe atomic ratio** |
| --- | --- | --- |
| Pristine ( α-Fe_2_O_3_ ) | 0.0015 | - |
| Sn (4 mol %)-Fe_2_O_3_ | 0.0115 | - |
| Be(6 mol %)-Fe_2_O_3_ | 0.0015 | 0.0052 |
| Sn (4 mol %)&Be (6 mol %)-Fe_2_O_3_ | 0.0091 | 0.0068 |

**Table S2.** Atomic percent of elements in as-prepared hematite samples was obtained by XPS.

| **Samples** | **Fe (At %)** | **Sn (At %)** | **O (At %)** | **Be (At %)** |
| --- | --- | --- | --- | --- |
| **α-Fe_2_O_3_** | 39.4 | 0.9 | 59.7 | - |
| **Sn-α-Fe_2_O_3_** | 37.7 | 2.3 | 60.1 | - |
| **Be-α-Fe_2_O_3_** | 39.8 | 0.6 | 56.3 | 3.3 |
| **Sn-Be-α-Fe_2_O_3_** | 36.0 | 2.6 | 57.1 | 4.4 |

**Table S3.** Conductivity from Hall Effect measurements for pristine, Sn-doped and co-doped α–Fe_2_O_3_ photoanodes sintered at 800°C.

| **Sample** | **Resistivity**  **(Ω.cm)** | **Conductivity**  **(1/Ω.cm)** |
| --- | --- | --- |
| **Pristine α–Fe_2_O_3_** | 2.63×10^-3^ | 3.81×10^2^ |
| **Sn-Doped α–Fe_2_O_3_** | 1.48×10^-3^ | 6.75×10^2^ |
| **Co-Doped α–Fe_2_O_3_** | 1.28×10^-3^ | 7.78×10^2^ |

**Table S4.** Output of the Equivalent Circuit Model for the of pristine, Sn-doped and co-doped α–Fe_2_O_3_ photoanodes at 1.23 V_RHE_, using 1M NaOH, under 1 sun standard illumination conditions from the Nyquist plot.

| **(R/Ω)**  **(CPE/F)** | **Pristine α-Fe_2_O_3_** | **Sn-doped α-Fe_2_O_3_** | **Co-doped α-Fe_2_O_3_** |
| --- | --- | --- | --- |
| **R_S_** | 36.4 | 21.2 | 13.3 |
| **R_CT1_**  **CPE_1_** | 133.1  3.21 x 10^-9^ | 75.1  1.05 x 10^-8^ | 48.3  2.67 x 10^-8^ |
| **R_CT2_**  **CPE_2_** | 198.7  2.98 x 10^-5^ | 113.8  3.56 x 10^-5^ | 86.9  4.65 x 10^-5^ |
